# Resolution of peritoneal metastases in CLDN18.2-positive gastric cancer treated with CAPOX plus zolbetuximab

**DOI:** 10.1093/jjco/hyag052

**Published:** 2026-03-31

**Authors:** Hironori Midorikawa, Nobukazu Hokamura, Taketoshi Fukasawa, Takeo Fukagawa

**Affiliations:** Department of Surgery, Teikyo University School of Medicine, 2-11-1 Kaga, Itabashi-ku, Tokyo 173-8606, Japan; Department of Surgery, Teikyo University School of Medicine, 2-11-1 Kaga, Itabashi-ku, Tokyo 173-8606, Japan; Department of Surgery, Teikyo University School of Medicine, 2-11-1 Kaga, Itabashi-ku, Tokyo 173-8606, Japan; Department of Surgery, Teikyo University School of Medicine, 2-11-1 Kaga, Itabashi-ku, Tokyo 173-8606, Japan

A man in his seventies presented with abdominal distension. Endoscopy revealed a type 3 tumor in the upper gastric body, and biopsy confirmed adenocarcinoma (Fig. 1). Staging laparoscopy demonstrated multiple peritoneal nodules with positive cytology (CY1), consistent with cT4aN0M1P1CY1 (Stage IVB) (Fig. 2). Systemic chemotherapy based on biomarker status is the standard treatment for stage IV gastric cancer with peritoneal dissemination [1]. Biomarker testing showed HER2 negativity, CLDN18.2 positivity, CPS ≥1, and microsatellite stability. CLDN18.2 expression was confirmed in both the primary tumor and peritoneal lesions.

**Figure 1:**
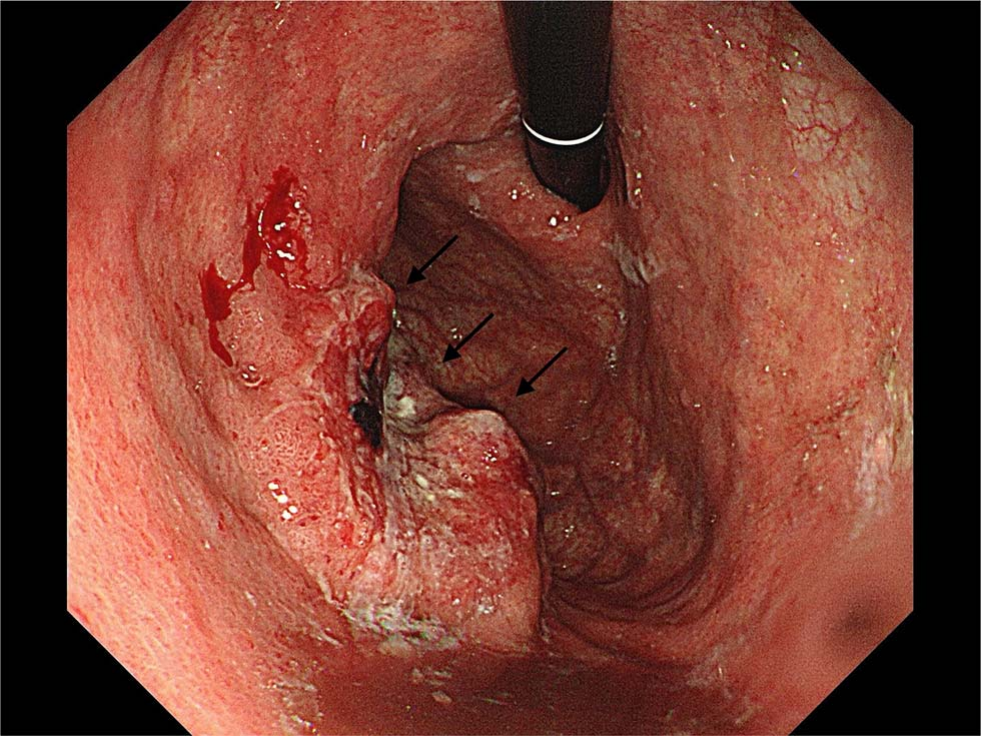
Endoscopic image before treatment showing a type 3 tumor in the upper gastric body.

**Figure 2:**
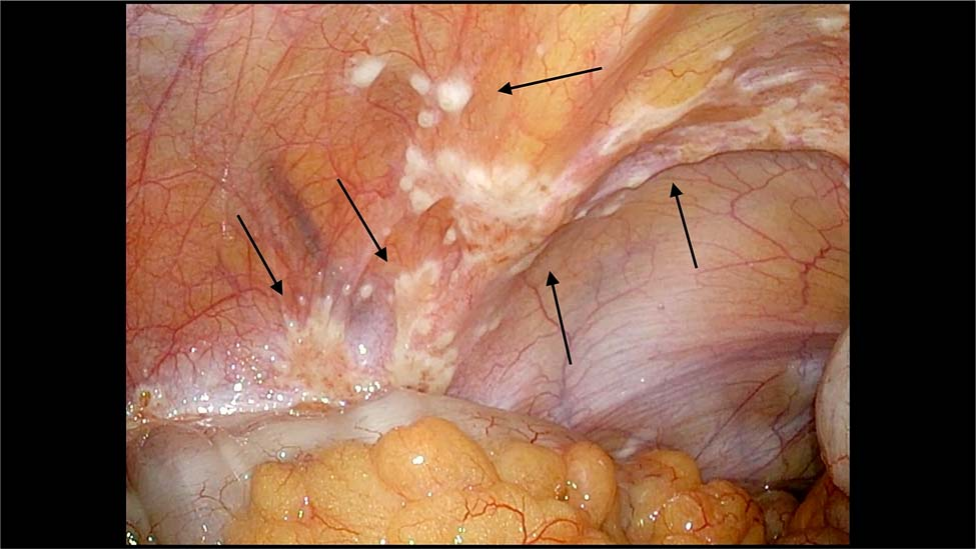
Staging laparoscopy before treatment demonstrating peritoneal dissemination in the rectovesical pouch.

First-line CAPOX combined with zolbetuximab was initiated following the regimen used in the GLOW trial [2], with zolbetuximab administered on Day 1 of each 3-week cycle. Three peritoneal nodules were biopsied at baseline, confirming metastatic adenocarcinoma. No grade ≥ 3 adverse events occurred during treatment.

After three cycles, endoscopy demonstrated marked shrinkage of the primary tumor (Fig. 3). Repeat staging laparoscopy after four cycles showed scarring of prior peritoneal lesions with no visible tumor (Fig. 4). Biopsy of scar tissue and repeat cytology were negative, confirming resolution of peritoneal metastases. The patient continues systemic therapy and remains under evaluation.

**Figure 3:**
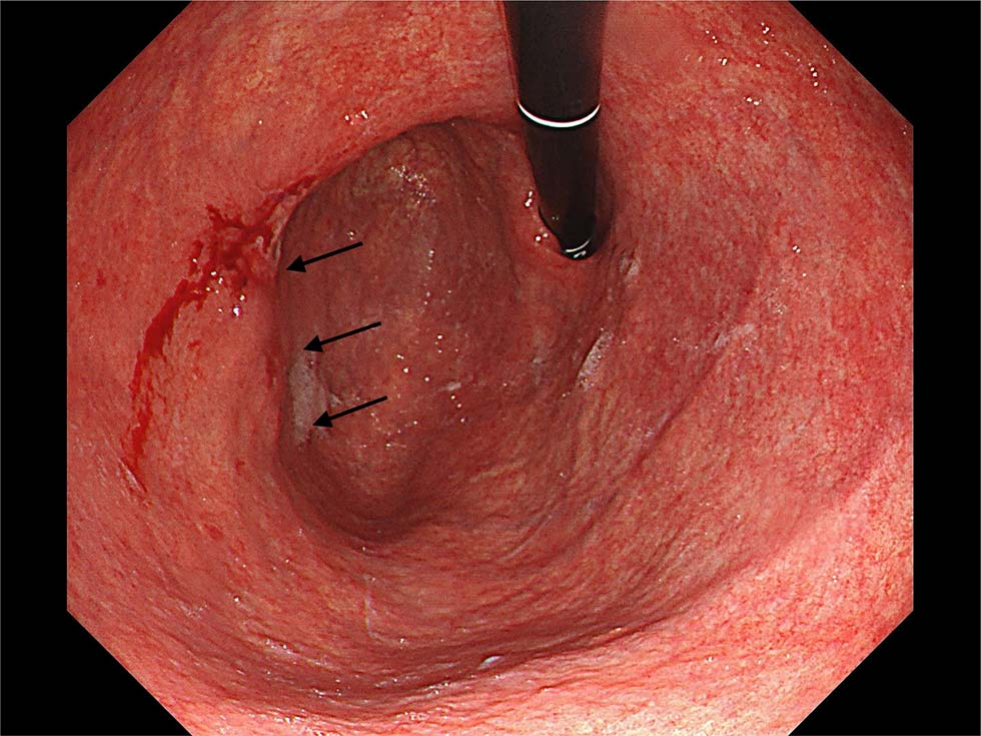
Endoscopic image after treatment demonstrating marked tumor regression.

**Figure 4:**
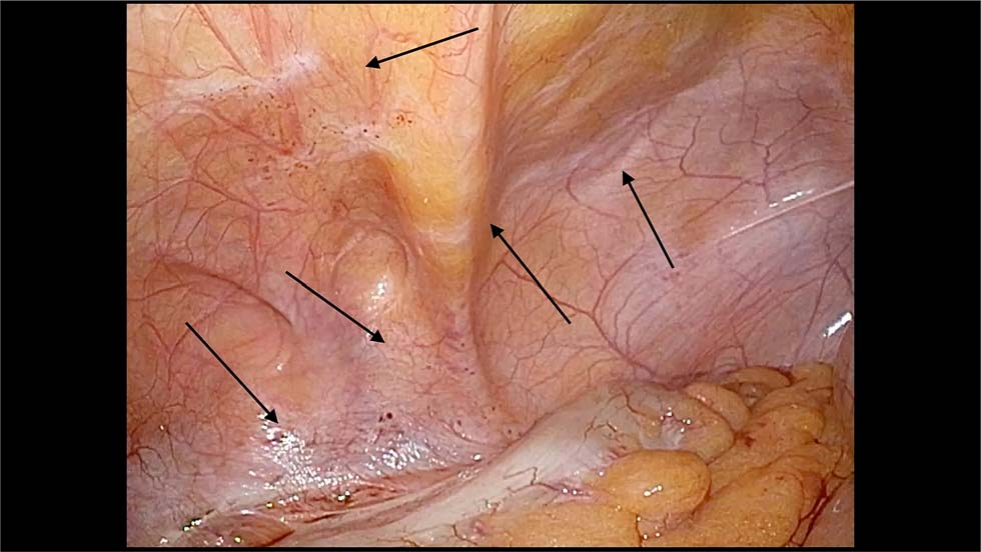
Staging laparoscopy after treatment revealing disappearance of peritoneal lesions in the rectovesical pouch.

Published evidence of complete disappearance of peritoneal metastases in CLDN18.2-positive gastric cancer treated with zolbetuximab-based chemotherapy is extremely limited, and prior phase III trials have not reported detailed rates of peritoneal disease resolution. This case is notable because treatment response was assessed not only by imaging but also by repeated laparoscopic and pathological evaluation, demonstrating sustained resolution of peritoneal dissemination in a biomarker-selected patient.

## References

[1] Japanese Gastric Cancer Association . Japanese gastric cancer treatment guidelines 2025 (7th edition). Gastric Cancer 2026;29:271–99. 10.1007/s10120-025-01698-4. 41569370 PMC12956939

[2] Shah MA, Shitara K, Ajani JA., et al. Zolbetuximab plus CAPOX in CLDN18.2-positive gastric or gastroesophageal junction adenocarcinoma: the randomized, phase 3 GLOW trial. Nat Med 2023;29:2133–41. 10.1038/s41591-023-02465-7. 37524953 PMC10427418

